# Supplementary material for: Canary Bornavirus (Orthobornavirus serini) Infections Are Associated with Clinical Symptoms in Common Canaries (Serinus canaria dom.)
Source: Viruses. 2022 Oct 4;14(10):2187. doi: 10.3390/v14102187 (PMC9607494; doi:10.3390/v14102187)
Supplement: Supplementary file 1 [file viruses-14-02187-s001.zip › viruses-1907900-supplementary.pdf]

Supplementary Materials

Table S1. Cross table for the number of canaries with positive or negative in **Macrorhabdus** tests as well as circovirus tests and occurrence of proventricular dilatation disease (PDD)-like signs. Expected counts for perfectly independent variables are given in brackets.

	Test status	PDD-like signs present	PDD-like signs absent	Total
Bornavirus positive[1]	Macrorhabdus positive	3 (3.4)	2 (1.6)	5
	Macrorhabdus negative	18 (17.6)	8 (8.4)	26
	Total	21	10	31
Bornavirus negative[2]	Macrorhabdus positive	7 (5.9)	21 (22.1)	28
	Macrorhabdus negative	18 (19.1)	73 (71.9)	91
	Total	25	94	119
All canaries[3]	Macrorhabdus positive	10 (10.1)	23 (22.9)	33
	Macrorhabdus negative	36 (35.9)	81 (81.1)	117
	Total	46	104	150

[1]p = 1.000 revealed by Fisher's exact test; [2]p = 0.599, revealed by chi-square test; [3]p = 0.959 in *all tested* birds, revealed by chi-square test

Table S2. Cross table for the number of canaries with positive or negative in **other yeasts (others than Macrorhabdus)** tests as well as circovirus tests and occurrence of proventricular dilatation disease (PDD)-like signs. Expected counts for perfectly independent variables are given in brackets.

	Test status	PDD-like signs present	PDD-like signs absent	Total
Bornavirus positive[1]	Other yeasts positive	3 (2.1)	0 (.9)	3
	Other yeasts negative	17 (17.9)	8 (7.1)	25
	Total	20	8	28
Bornavirus negative[2]	Other yeasts positive	7 (6.2)	23 (23.8)	30
	Other yeasts negative	17 (17.8)	70 (69.2)	87
	Total	24	93	119
All canaries[3]	Other yeasts positive	10 (10.0)	23 (22.9)	33
	Other yeasts negative	34 (34.0)	78 (78.0)	112
	Total	44	101	145

[1]p = .536 revealed by Fisher's exact test; [2]p = .657 revealed by chi-square test; [3]p = .995 revealed by chi-square test

Table S3. Cross table for the number of canaries with positive or negative **in trichomonads** tests as well as circovirus tests and occurrence of proventricular dilatation disease (PDD)-like signs. Expected counts for perfectly independent variables are given in brackets.

	**Test status**	**PDD-like signs present**	**PDD-like signs absent**	**Total**
Bornavirus positive[1]	Trichomonads positive	2 (2.0)	1 (1.0)	3
	Trichomonads negative	10 (10.0)	5 (5.0)	15
	Total	12	6	18
Bornavirus negative[2]	Trichomonads positive	5 (5.9)	20 (19.4)	25
	Trichomonads negative	16 (15.1)	48 (48.9)	64
	Total	21	68	89
All canaries[3]	Trichomonads positive	7 (8.6)	21 (19.4)	28
	Trichomonads negative	26 (24.4)	53 (54.6)	79
	Total	33	74	107

[1]p = 1.000 revealed by Fisher's exact test; [2]p = *0.618* revealed by chi-square test; [3]p = *0.485* revealed by chi-square test).

Table S4. Multivariate linear regression analysis (dependent variable: PDD-signs) using Model 1. predictors: bornavirus PCR and Model 2. bornavirus PCR, source (live clinic patient vs. dead bird).

		unstandardized coefficients		standardized coefficients		
Model		B	Standard error	Beta	t	p-value
1[1]	(constant)	0.223	0.034		6.567	0.000
	bornavirus PCR	0.495	0.076	0.423	6.505	0.000
	Adjusted-R^2	0.175				
2[2]	(constant)	0.207	0.049		4.189	0.000
	Bornavirus PCR	0.503	0.078	0.430	6.440	0.000
	Source	0.028	0.062	0.030	0.453	0.651
	Adjusted-R^2	0.171				

[1]Adjusted-R^2 0.175 [2]Adjusted R^2 0.171
